# Corrigendum

**DOI:** 10.1002/rcr2.455

**Published:** 2019-06-26

The authors would like to draw the reader's attention to an error in the following article:

Tomioka, H. and Takada, H. (2017) Treatment with nintedanib for acute exacerbation of idiopathic pulmonary fibrosis. *Respirology Case Reports*, 5 (2), e00215. https://doi.org/10.1002/rcr2.215.

Dr Hirohito Takada's last name was misspelt.

The correct spelling is: Hirohito Takata.

The authors apologize for this error and any confusion it may have caused.

